# Supplementary material for: Energy storing bricks for stationary PEDOT supercapacitors
Source: Nat Commun. 2020 Aug 11;11:3882. doi: 10.1038/s41467-020-17708-1 (PMC7419536; doi:10.1038/s41467-020-17708-1)
Supplement: Supplementary file 1 — Supplementary Information [file 41467_2020_17708_MOESM1_ESM.pdf]

Supplementary Materials for

Energy Storing Bricks for Stationary PEDOT Supercapacitors

Hongmin Wang[1], Yifan Diao[2], Yang Lu[2], Haoru Yang[1], Qingjun Zhou[2], Kenneth Chrulski[1] and Julio M. D'Arcy[1,2]*

Correspondence to: jdarcy@wustl.edu

Supplementary Figures

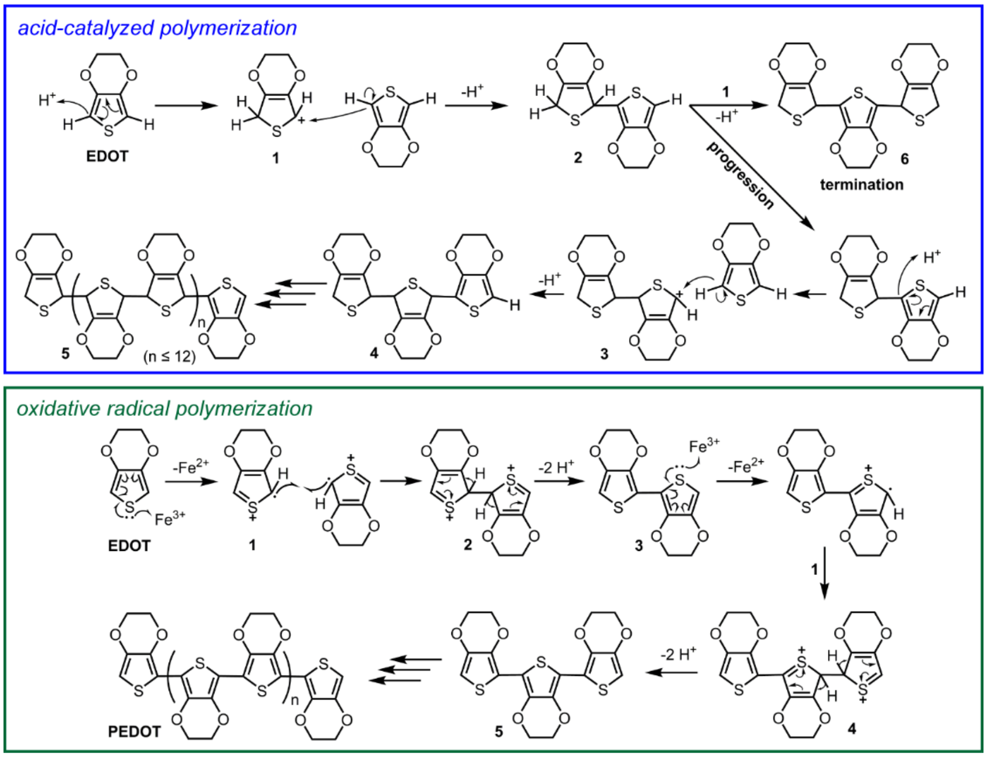

Supplementary Figure 1 | Polymerization mechanisms of EDOT. Both acid and Fe^{3+} serve as initiators for the polymerization of EDOT. Acid leads to acid-catalyzed polymerization producing oligomers whereas Fe^{3+} results in oxidative radical polymerization generating PEDOT. In acid-catalyzed polymerization (blue box), EDOT is protonated forming a cation (1), then combines with another monomer and is deprotonated resulting in a dimer (2). The dimer is further protonated (3) and polymerized leading to trimer (4) and oligomers (5). The degree of polymerization is $n \leq 12$ because of active chain termination (6). In oxidative radical polymerization (green box), EDOT is oxidized by Fe^{3+} to form a radical cation (1); two radical cations (1) combine to form a dimer cation (2) that undergoes deprotonation leading to a neutral dimer (3). The dimer is further oxidized and polymerized forming protonated trimer cation (4), neutral trimer (5) and PEDOT.

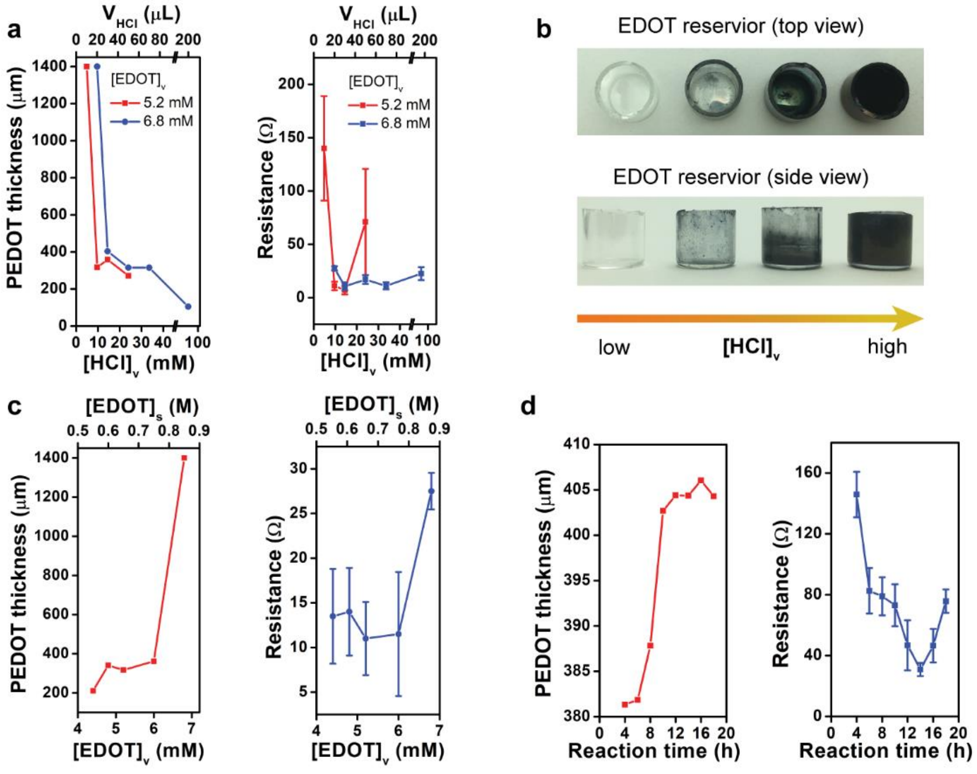

Supplementary Figure 2 | Synthesis of a PEDOT coating on the surface of a brick. (**a**) Acid concentration ([HCl]) determines the reaction pathway as demonstrated by control of coating thickness and electrical resistance. These experiments are carried out using EDOT vapor concentrations of 5.2 mM and 6.8 mM. We calculate vapor concentration $[HCl]_v$ from the volume of concentrated HCl (V_{HCl}) and by assuming complete HCl evaporation. A lower $[HCl]_v$ increases PEDOT coating thickness because acid-catalyzed polymerization is minimized. Thicker coatings are produced by holding $[HCl]_v$ constant while increasing EDOT concentration. The lowest electrical resistance is obtained using 14 mM $[HCl]_v$ because this concentration promotes both steady-state dissolution rate and oxidative radical polymerization. (**b**) A high concentration of HCl leads to acid-catalyzed polymerization and uncontrolled polymerization that results in oligomers of dark color. Photographs show coatings on monomer reservoirs that formed in situ during the reaction. (**c**) Plot of a PEDOT coating's thickness versus electrical resistance collected at different

EDOT concentrations ($[EDOT]_v$) and using $[HCl]_v$ = 9.6 mM. A higher $[EDOT]_v$ increases the coating thickness however also leads to higher resistance. In this plot, $[EDOT]_s$ = concentration of EDOT in chlorobenzene solution, and $[EDOT]_v$ = EDOT vapor concentration (calculated assuming all the EDOT evaporates). (**d**) Time-dependent plots of reactions for quenching experiments show the thickness of a PEDOT coating versus electrical resistance.

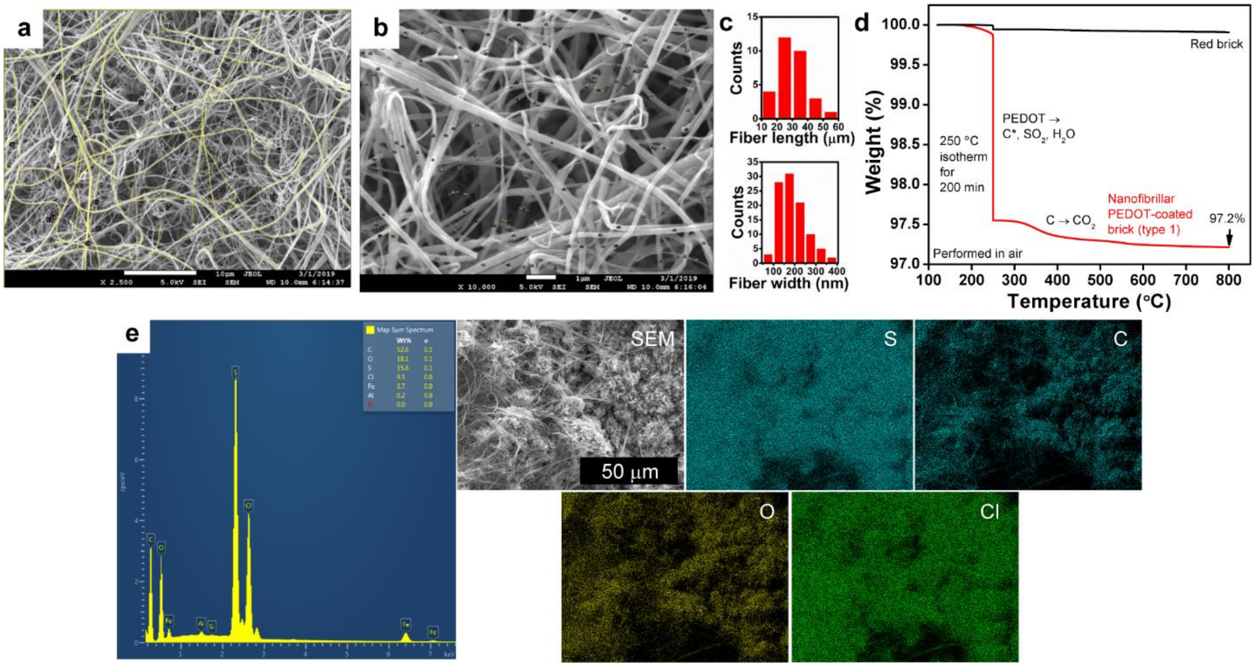

Supplementary Figure 3 | Electron microscopy, thermogravimetric analysis and energy dispersive spectroscopy of a PEDOT-coated brick. Scanning electron micrograph shows nanofibers and ImageJ software is used for calculating (**a**) length and (**b**) width of nanofibers. (**c**) Histograms show the results of dimensional analysis. (**d**) Thermogravimetric analysis of brick and PEDOT-coated brick shows that the latter contains a 2.8 wt% mass loading of polymer. (**e**) Energy-dispersive X-ray spectra and maps of a purified PEDOT coating show C, O, S and Cl signals pertaining to a Cl^- doped PEDOT structure; elemental ratios are non-stoichiometric due to brick's impurities.

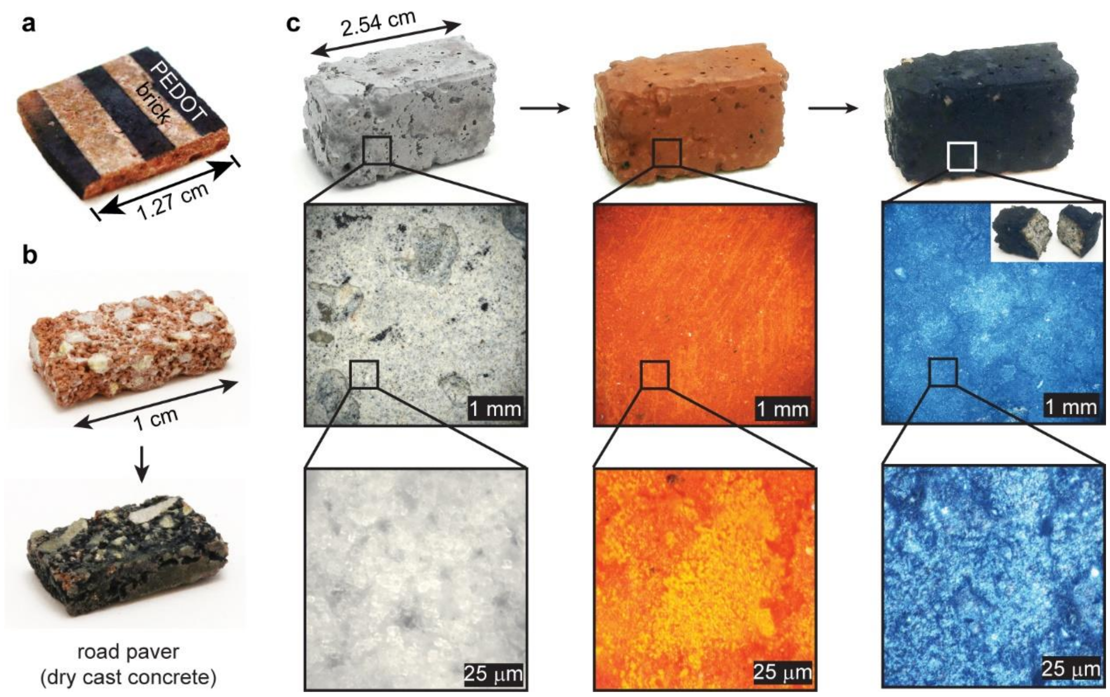

Supplementary Figure 4 | PEDOT coatings on patterned brick and concretes. (**a**) A tape mask produces a PEDOT-patterned type 1 brick during synthesis. (**b**) PEDOT coats a red-colored road paver made of dry cast concrete; the heterogeneous surface is due to large gravel sites that are inert during the reaction. (**c**) A bar of concrete (left column) is coated with α-Fe_2O_3 particles (middle column) via dip coating in an aqueous dispersion and used for synthesis, resulting in PEDOT-coated concrete (right column). This composite exhibits a coating with a 1 kΩ two-point probe resistance.

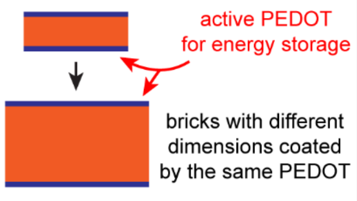

Parameters	Volume	Total mass	PEDOT mass	Active area	Gravimetric capacitance	Volumetric capacitance	Areal capacitance
Constant?	N	N	N	N	N	N	Y

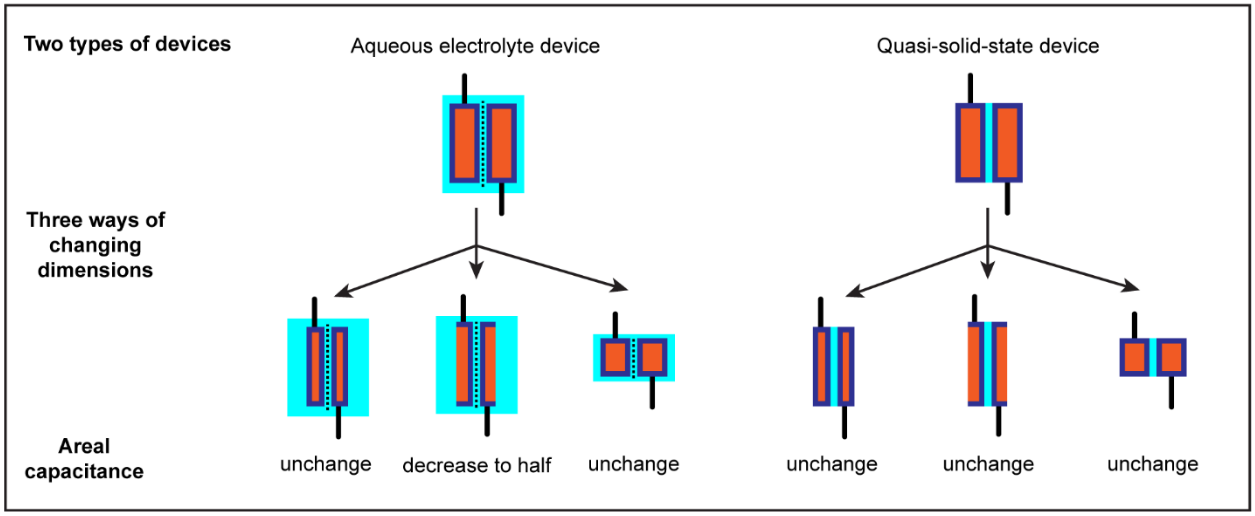

Supplementary Figure 5 | Evaluation of electrode/device metrics affected by brick dimensions. The upper table shows that when a PEDOT-coated brick electrode changes dimension, only areal metrics parameters provide a valid approach for assessing a PEDOT coating quality and thickness. Other normalizing parameters based on mass or volume change with brick dimensions. The lower box shows the change of areal capacitance with respect to supercapacitor dimensions; device design is based on two types of devices using 1) aqueous electrolyte or 2) gel electrolyte (quasi-solid-state). Device dimensions are tailored using bricks of various thicknesses and lengths and by tailoring the active surface area i.e., cutting off a section of a PEDOT-coated brick using a diamond saw. For an aqueous electrolyte-based device, capacitance remains constant regardless of brick dimension however cutting a brick decreases areal capacitance; removing half of the coated area in contact with electrolyte leads to 50% lowering of capacitance. For a quasi-solid-state device, the areal capacitance is unaffected by device dimension because only one face on each brick electrode is accessible to the electrolyte.

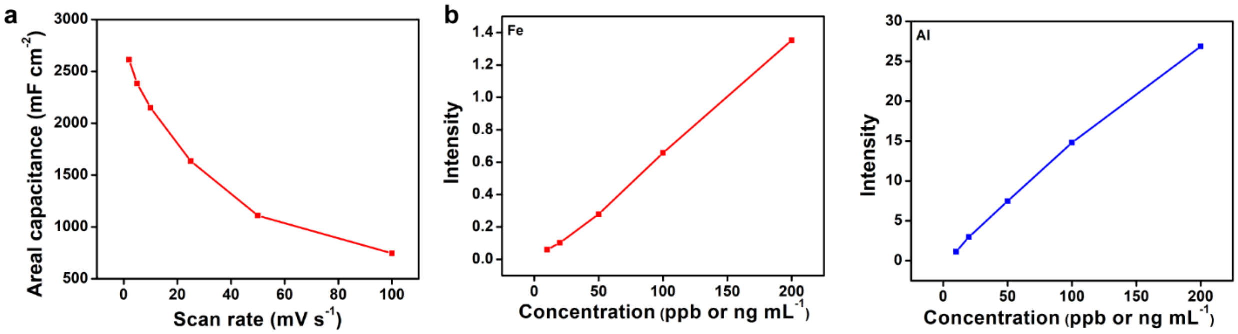

c

	Blank 1 M H_2SO_4	Electrolyte after cycling				
	Detected conc. (1/100 diluted, ng mL^{-1})		Original conc. (ng mL^{-1})	Total mass in 5 mL electrolyte (mg)	Equivalent oxide mass (mg)	Oxide wt% of the electrode (%)
**Fe**	2.700	47.066	4436.6	0.02218	0.03163	0.0127
**Al**	0.081	19.785	1970.4	0.00985	0.01862	0.0075

Supplementary Figure 6 | Three-electrode rate capability of PEDOT-coated brick electrodes and inductively coupled plasma mass spectrometry of electrolytes. (**a**) Areal capacitances of the electrode at different scan rates via cyclic voltammetry. (**b**) External calibration curves of Fe and Al for inductively coupled plasma mass spectrometry analysis. (**c**) Summary of concentrations obtained from 1 M H_2SO_4 electrolyte after cycling the brick electrode at 2, 5, 10, 25, 50 and 100 mV s^{-1} for 10 cycles each shows negligible α-Fe_2O_3 and Al_2O_3 dissolution.

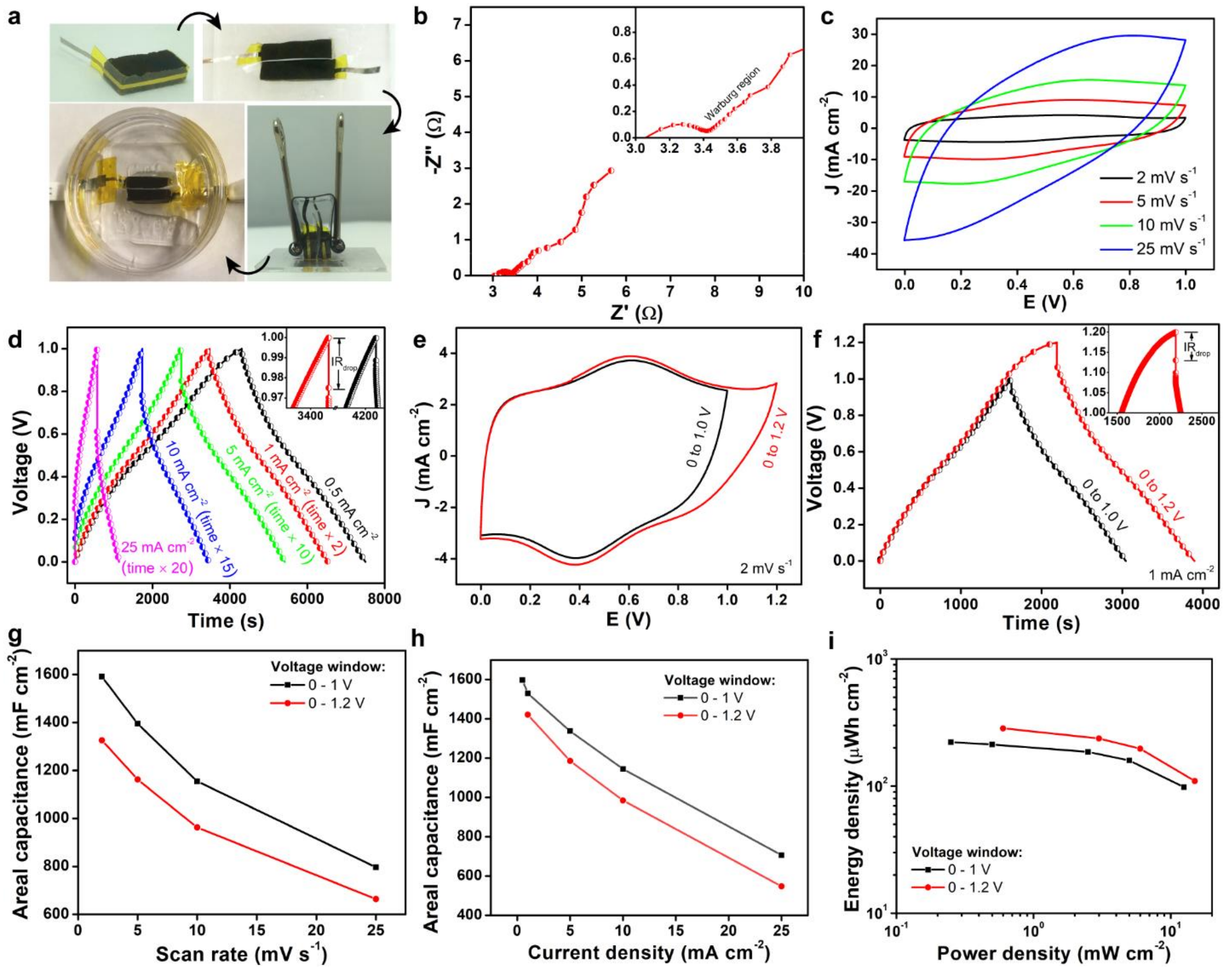

Supplementary Figure 7 | Electrochemical characterization of PEDOT-coated brick supercapacitor in aqueous electrolyte. (**a**) Step-by-step fabrication of an aqueous-electrolyte based supercapacitor where a 1 cm × 0.5 cm × 0.28 cm electrode is fabricated by attaching a Pt wire to a PEDOT-coated brick using polyimide tape. A polymer separator is sandwiched between two electrodes held together using a binder clip and epoxy (the binder clip is removed once the epoxy cures). Left bottom shows a top view of a supercapacitor after epoxy cures; the supercapacitor is placed in a petri dish containing 1 M H_2SO_4 electrolyte. (**b**) Nyquist plot of supercapacitor shows a Warburg region and internal resistance of 3 Ω. (**c**) Cyclic voltammograms for a supercapacitor collected at scan rates ranging from 2 to 25 mV s^{-1}. (**d**) Galvanostatic charge-

discharge profiles for supercapacitor at current densities ranging between 0.5 and 25 mA cm^{-2} (inset shows IR drop at 0.5 and 1 mA cm^{-2}). (**e**) Cyclic voltammograms and (**f**) galvanostatic charge-discharge profiles retain the shape of the curve at voltage windows of 1 V and 1.2 V. Areal capacitance at different (**g**) scan rates from cyclic voltammetry and (**h**) current densities from galvanostatic charge-discharge measurements with 1 V and 1.2 V voltage window. (**i**) Ragone plot with 1 V and 1.2 V voltage windows.

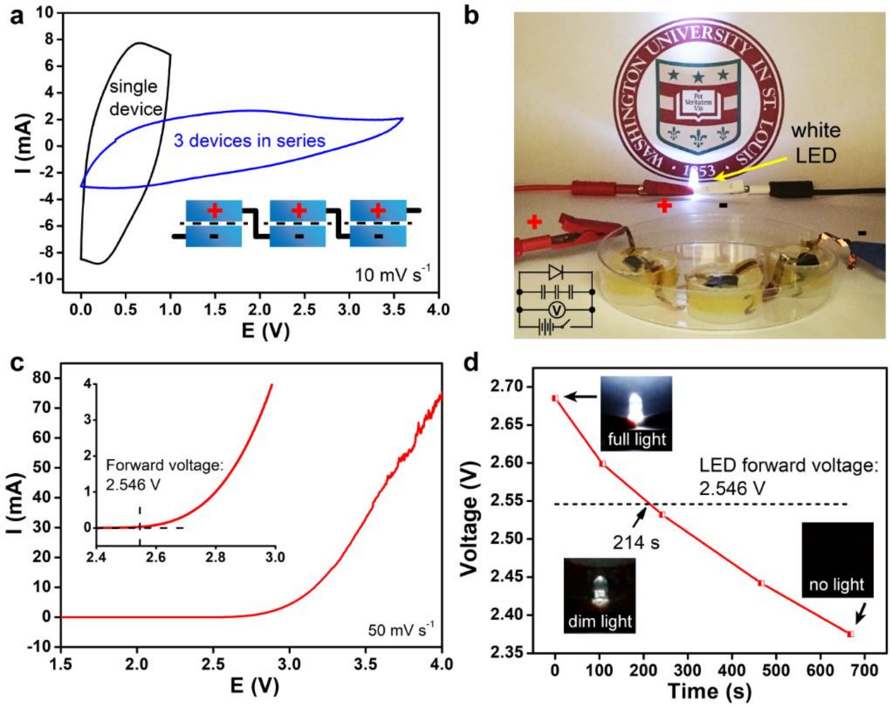

Supplementary Figure 8 | Electrochemical characterization of a tandem device comprised of three supercapacitors connected in series. (**a**) Cyclic voltammograms for a single device versus tandem device at 10 mV s^{-1}. (**b**) The supercapacitor lights a white light-emitting diode with (**c**) forward voltage ("turn-on" voltage) of 2.546 V. (**d**) Discharging profile of supercapacitor during the lighting of a light-emitting diode shows a decrease in voltage from 2.7 V to 2.4 V in 10 min with diminishing light intensity (see Supplementary Movie).

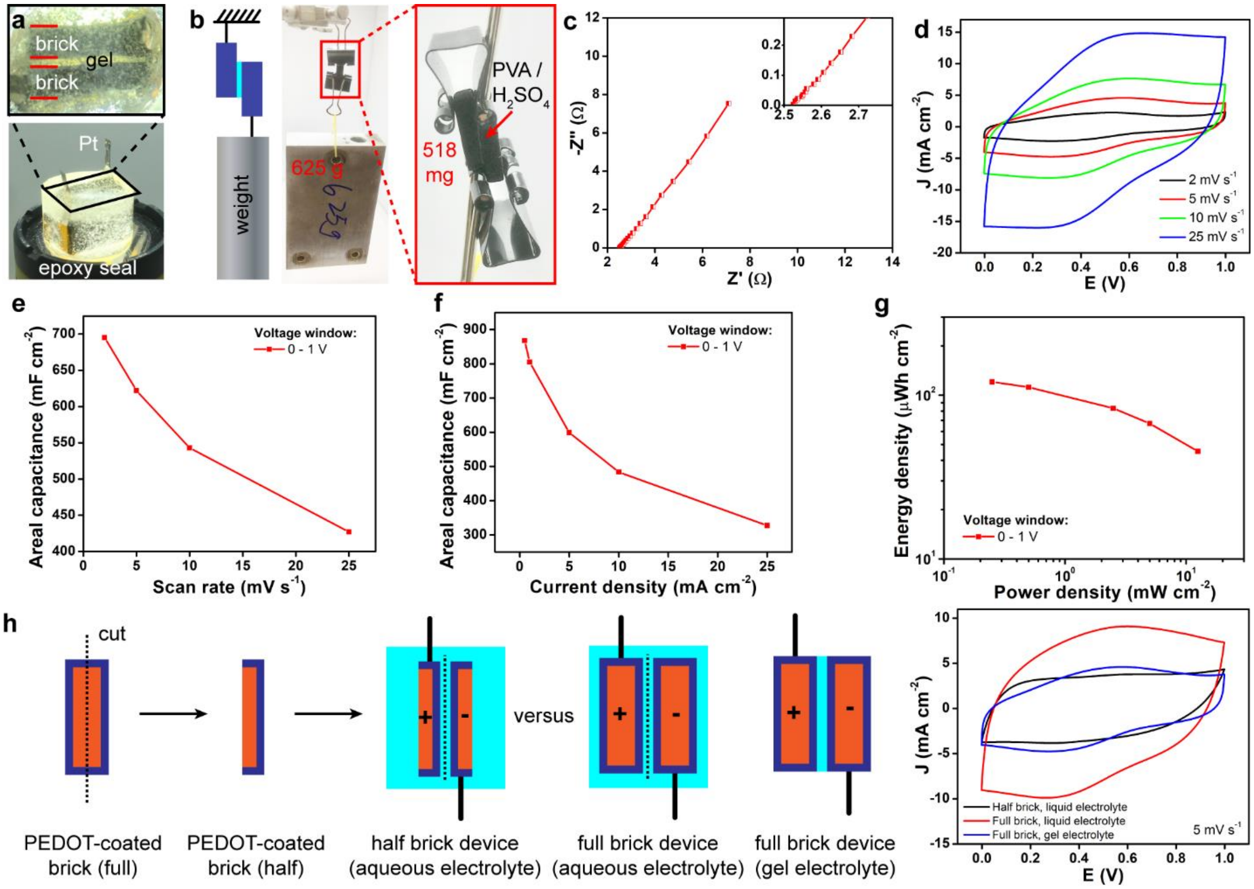

Supplementary Figure 9 | Quasi-solid-state supercapacitor based on PEDOT-coated brick electrodes. (**a**) Supercapacitor is comprised of two electrodes (1 cm × 0.5 cm × 0.28 cm) and a poly(vinyl alcohol)/H_2SO_4 gel electrolyte layer (0.7 mm thick); epoxy seals the entire device in a cylindrical reservoir (puck). (**b**) The brick-gel-brick sandwich structure (518 mg) withstands a shearing force more than 1000 times its own weight – this is carried out by attaching an aluminum block (625 g) to the electrode and by allowing gravity to pull on it. (**c**) Nyquist plot shows an internal resistance of 2.5 Ω and the absence of semicircle associated with charge transfer resistance. (**d**) Cyclic voltammogram at scan rates ranging from 2 to 25 mV s^{-1}. Areal capacitance at different (**e**) scan rates from cyclic voltammetry and (**f**) current densities from galvanostatic charge-discharge measurements with 1 V voltage window. (**g**) Ragone plot shows the device energy density at different power densities with 1 V voltage window. (**h**) Cutting brick electrodes

in half removes half of the PEDOT coating resulting in a 50% decrease in the areal capacitance when using an aqueous electrolyte (calculated from cyclic voltammograms at a scan rate of 5 mV s^{-1}). This 50% lower magnitude in capacitance is similar to that of a quasi-solid-state device fabricated using fully coated bricks because gel permeation is limited to a single face.

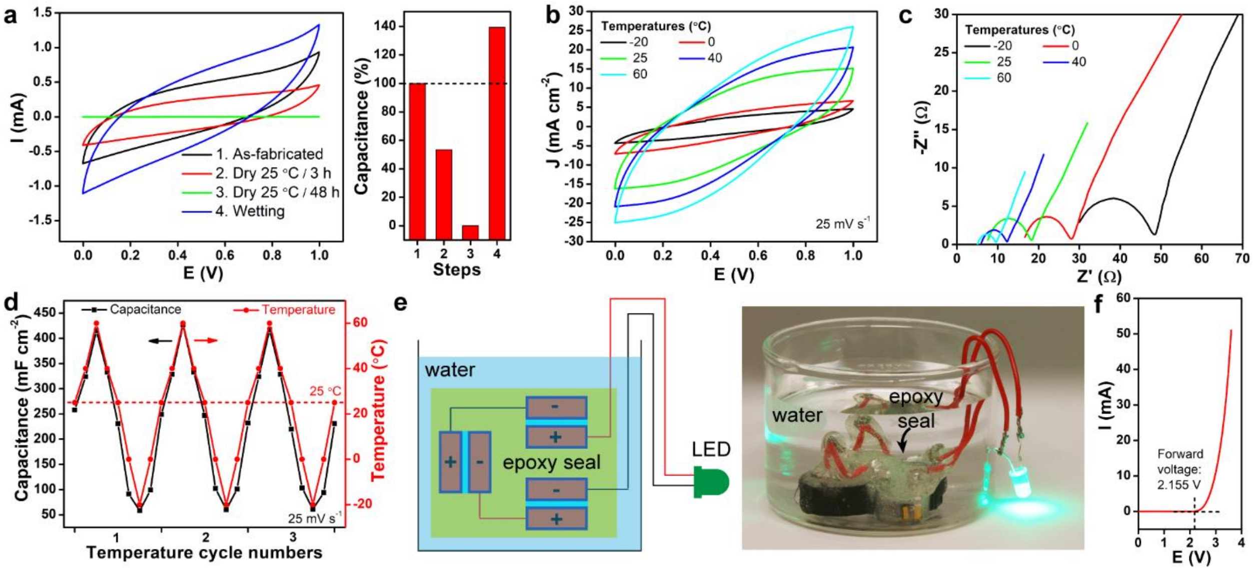

Supplementary Figure 10 | Environmental performance of quasi-solid-state supercapacitor based on PEDOT-coated brick electrodes. (**a**) Cyclic voltammogram of unsealed supercapacitor shows continuous capacitance degradation at room temperature due to solvent evaporation as indicated by a decreasing curve area. Histogram (right) shows capacitance recovery after wetting (blue line); note that curve area increases beyond original capacitance due to the creation of a liquid ionic percolation network after wetting. (**b**) Cyclic voltammograms and (**c**) Nyquist plots, collected during heating-cooling cycles between -20 °C and 60 °C, show that (**d**) capacitance is temperature-dependent and reversible. (**e**) After sealed by epoxy, a tandem device fabricated by connecting three quasi-solid supercapacitors in series lights up a green light-emitting diode with (**f**) a forward voltage of 2.155 V when submerged underwater.

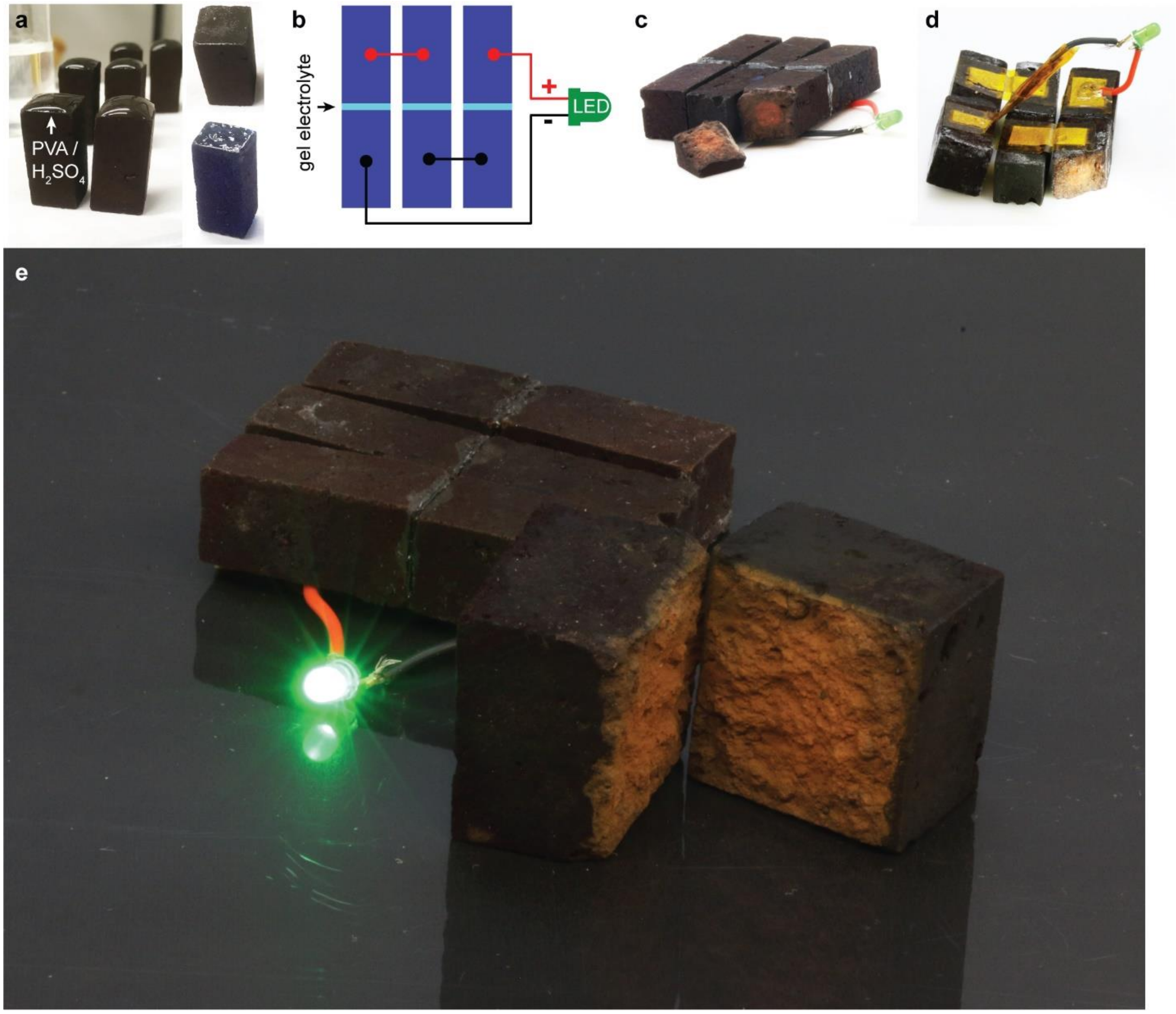

Supplementary Figure 11 | Scale-up of a quasi-solid-state supercapacitor based on PEDOT-coated brick electrodes. (**a**) Casting of poly(vinyl alcohol)/H_2SO_4 gel electrolyte on large PEDOT-coated brick electrodes (2 × 1 × 1 cm). Photographs show casting of electrolyte and permeation into brick resulting in a dry thin film (right-top). A second casting of gel electrolyte, followed by drying, results in a thick gel electrolyte layer (right bottom). (**b**) Electrical circuit diagram of three supercapacitors connected in series used for lighting up a light-emitting diode; dark blue rectangles represent PEDOT-coated bricks and turquoise color represents the gel electrolyte. Digital photographs show the (**c**) front and the (**d**) back of a quasi-solid-state

supercapacitor module. (**e**) Raw photographs (post-processing omitted) show 1) tandem device lighting up a green light-emitting diode and 2) the core-shell architecture of a nanofibrillar PEDOT-coated brick electrode.

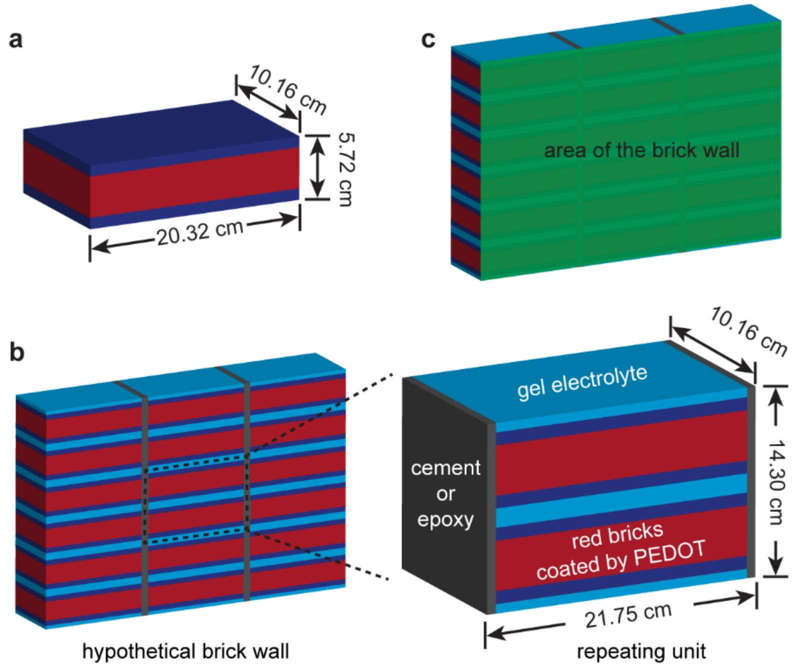

Supplementary Figure 12 | Estimation of capacitance and energy density delivered by a hypothetical brick wall. (**a**) Each brick (20.32 cm × 10.16 cm × 5.72 cm or 8 inches × 4 inches × 2.25 inches) contains two 20.32 cm × 10.16 cm faces coated by nanofibrillar PEDOT. In the illustration, red color represents red brick and dark blue color represents PEDOT. (**b**) A brick wall is constructed using 1.43 cm-thick gel electrolyte (1/4 of the brick height, represented in light blue color) and 1.43 cm-thick cement (or epoxy, represented in grey color). This wall contains repeating supercapacitor units with a dimension of 21.75 cm × 10.16 cm × 14.30 cm (comprised of two bricks stacked together). (**c**) The front wall area is labeled in green color and our calculations (based on PEDOT-coated type 1 brick results) show that this hypothetical brick wall provides a maximum capacitance of 11.5 kF m^{-2} and an energy density of 1.61 Wh m^{-2} (Supplementary Discussion).

Supplementary Tables

Supplementary Table 1. Geometries and mass loadings of electrodes and devices

Electrodes/ devices	Size (cm)	Volume (cm^3)	A (cm^2)	$m_{electrode}$ (mg)	m_{device} (mg)	PEDOT mass loading (wt%)	m_{PEDOT} (mg)
Three-electrode characterization	1×0.5×0.28	0.14	0.5	249	-	2.8	6.97
Aqueous electrolyte supercapacitor	1×0.5×0.5625	0.28	0.5	-	499	2.8	13.94
Quasi-solid-state supercapacitor	1×0.5×0.63	0.32	0.5	-	518	2.7	13.94

Note: A is the active electrode area i.e., the area in contact with electrolyte. In both three-electrode characterization and quasi-solid-state supercapacitor, A = 0.5 cm^2. For aqueous electrolyte supercapacitor, we take the electrode area directly in contact with separator as the active electrode area i.e., A = 0.5 cm^2. Note that in an aqueous electrolyte supercapacitor, PEDOT on side faces may also contribute to the capacitance thus areal metrics for this device are qualitative.

Supplementary Table 2. Areal, gravimetric and volumetric metrics of electrodes and devices

Electrodes/ Devices	C_a (F cm^{-2})	$C_{g(PEDOT)}$ (F g^{-1})	$C_{g(brick\ or\ device)}$ (F g^{-1})	C_v (F cm^{-3})	E_a (μWh cm^{-2})	P_a (mW cm^{-2})	E_v (μWh cm^{-3})	P_v (mW cm^{-3})
Three-electrode characterization	2.61 (2 mV s^{-1})	187 (2 mV s^{-1})	5.24 (2 mV s^{-1})	9.33 (2 mV s^{-1})	-	-	-	-
Aqueous electrolyte supercapacitor	1.59 (2 mV s^{-1}, CV)	56.9 (2 mV s^{-1}, CV)	1.59 (2 mV s^{-1}, CV)	2.83 (2 mV s^{-1}, CV)	-	-	-	-
Aqueous electrolyte supercapacitor	1.60 (0.5 mA cm^{-2}, GCD)	57.2 (0.5 mA cm^{-2}, GCD)	1.60 (0.5 mA cm^{-2}, GCD)	2.84 (0.5 mA cm^{-2}, GCD)	222 (0.5 mA cm^{-2}, GCD)	0.25 (0.5 mA cm^{-2}, GCD)	394 (0.5 mA cm^{-2}, GCD)	0.44 (0.5 mA cm^{-2}, GCD)
Quasi-solid-state supercapacitor	0.868 (0.5 mA cm^{-2}, GCD)	31.0 (0.5 mA cm^{-2}, GCD)	0.87 (0.5 mA cm^{-2}, GCD)	1.38 (0.5 mA cm^{-2}, GCD)	121 (0.5 mA cm^{-2}, GCD)	0.25 (0.5 mA cm^{-2}, GCD)	192 (0.5 mA cm^{-2}, GCD)	0.40 (0.5 mA cm^{-2}, GCD)

Supplementary Methods

Equations

The capacitance C (mF) of electrodes and devices are calculated using Supplementary Equation 1:

$$C = q\ V^{-1} \qquad (1)$$

Where q (mC) is the total amount of charge stored and V (V) is the potential change during discharge. In three-electrode characterization of a single electrode, q is calculated from cyclic voltammogram using Supplementary Equation 2:

$$q = \int_0^t I dt \qquad (2)$$

Where 0 and t (s) mark the time of beginning and end of a discharge cycle, and I (mA) is the discharge current.

In two-electrode characterization of the symmetric supercapacitor, q is calculated from galvanostatic charge-discharge curves using Supplementary Equation 3:

$$q = It \quad (3)$$

Where I (mA) is the discharge current, and t (s) is the discharge time.

The specific capacitance C_{sp} such as areal capacitance C_a (mF cm^{-2}), gravimetric capacitance C_g (F g^{-1}) and volumetric capacitance C_v (mF cm^{-3}) are calculated using Supplementary Equations 4 – 6:

$$C_a = C\ A^{-1} \qquad (4)$$

$$C_g = C\ m^{-1} \qquad (5)$$

$$C_v = C\ v^{-1} \qquad (6)$$

Where C (mF) is the capacitance, A (cm^2) is the active electrode area; m (mg) is the mass of the electrode, device, or active material; v (cm^3) is the volume of the electrode or device.

The areal energy density E_a (μWh cm^{-2}) of a symmetric supercapacitor is calculated from galvanostatic discharge curves using Supplementary Equation 7:

$$E_a = C_a V^2 (2\times3.6)^{-1} \quad (7)$$

Where C_a (mF cm^{-2}) is areal capacitance obtained from Supplementary Equation 4, and V (V) is the potential change during the discharge.

The areal power density P_a (mW cm^{-2}) of a symmetric supercapacitor is calculated from galvanostatic discharge curves using Supplementary Equation 8:

$$P_a = 3.6\ E_a\ t^{-1} \quad (8)$$

Where E_a is the areal energy density obtained from Supplementary Equation 7, and t is the discharge time (s). Volumetric energy density E_v (μWh cm^{-3}) and power density P_v (mW cm^{-3}) are calculated similarly using volumetric capacitance C_v.

Coulombic efficiency CE (%) of the symmetric supercapacitor is calculated using Supplementary Equation 9:

$$CE = (q_{discharge}\ q_{charge}^{-1})\times100\% \qquad (9)$$

Where $q_{discharge}$ (mC) is the amount of charge released from the supercapacitor during galvanostatic discharging, and q_{charge} (mC) is the amount of charge that entered the supercapacitor during galvanostatic charging. In galvanostatic charge-discharge measurements, the current I (mA) is a constant during charging and discharging thus by combining Supplementary Equation 3 and Supplementary Equation 9, we produce the simplified Supplementary Equation 10:

$$CE = (t_{discharge}\ t_{charge}^{-1})\times100\% \quad (10)$$

Where $t_{discharge}$ (s) is the discharge time and t_{charge} (s) is the charge time.

Three-electrode characterization calculation

The electrode structure is shown in Fig. 4a. Cyclic voltammogram is used to calculate capacitances and energy densities. The q in Supplementary Equation 2 is obtained from a 2 mV s^{-1} scan with 1 V voltage window (Fig. 4b) and equals 1306 mC (applying Supplementary Equation 1: C = 1306 mC / 1 V = 1306 mF). Using Supplementary Equations 4-6 and data from the first row of Supplementary Table 1, we obtain: C_a = 1306 mF / 0.5 cm^2 = 2.61 F cm^{-2}; $C_{g(brick)}$ = 1306 mF / 249 mg = 5.24 F g^{-1} based on the mass of the entire brick electrode; $C_{g(PEDOT)}$ = 1306 mF / (249 mg × 2.8%) = 187 F g^{-1} based on PEDOT mass; C_v = 1306 mF / (1 cm × 0.5 cm × 0.28 cm) = 9.33 F cm^{-3} based on the volume of the entire electrode. Metrics at higher scan rates (Supplementary Fig. 6a) are calculated using the same methods discussed above.

Aqueous electrolyte supercapacitor calculation

Device structure is shown in Fig. 5a upper row and Supplementary Figure 7a. Given that this proof-of-concept device is for electrode performance evaluation, we only consider the mass and dimension of brick electrodes and separator excluding Pt current lead, polyimide tape, epoxy, glass substrate and Petri dish. The thickness of separator membrane (Celgard 3501) is 0.0025 cm leading to a device thickness of 0.28 cm + 0.28 cm + 0.0025 cm = 0.5625 cm. The length and width of the device are the same as a single electrode (1 cm × 0.5 cm). The device mass is 249 mg + 249 mg + 0.9 mg = 499 mg (includes the mass of the separator = 0.9 mg). The mass of PEDOT in the device is 249 mg × 2.8% × 2 = 13.94 mg, the mass loading of PEDOT in the device is 13.94 mg / 499 mg × 100% = 2.8%. When calculating capacitance from cyclic voltammogram, q is obtained (795.6 mC) using a 2 mV s^{-1} scan rate (black curve in Fig. 5b and Supplementary Fig. 7c). Applying q to Supplementary Equation 1: C = 795.6 mC / 1 V = 795.6 mF. Using Supplementary Equations 4-6 and data from the second row of Supplementary Table 1 leads to: C_a = 795.6 mF / 0.5 cm^2 = 1.59 F cm^{-2}; $C_{g(brick)}$ = 795.6 mF / 499 mg = 1.59 F g^{-1} based on the mass of the device; $C_{g(PEDOT)}$ =

795.6 mF / (499 mg × 2.8%) = 56.9 F g^{-1} based on PEDOT mass; C_v = 795.6 mF / (1 cm × 0.5 cm × 0.5625 cm) = 2.83 F cm^{-3} based on the volume of the device. Galvanostatic charge-discharge measurement at a current density of 0.5 mA cm^{-2} (Supplementary Fig. 7d) is also used to calculate capacitance, as well as energy density and power density of the device. From the profile, the discharging time is 3194.1 s, leading to a q of 798.5 mC (obtained using Supplementary Equation 3: 0.5 mA cm^{-2} × 0.5 cm^2 × 3194.1s = 798.5 mC). Appling q to Supplementary Equation 1 results in C = 798.5 mC / 1 V = 798.5 mF. Using Supplementary Equations 4-6 and data from the second row of Supplementary Table 1 leads to: C_a = 798.5 mF / 0.5 cm^2 = 1.60 F cm^{-2}; $C_{g(device)}$ = 798.5 mF / 499 mg = 1.60 F g^{-1} based on the mass of the device; $C_{g(PEDOT)}$ = 798.5 mF / (499 mg × 2.8%) = 57.2 F g^{-1} based on PEDOT mass; C_v = 798.5 mF / (1 cm × 0.5 cm × 0.5625 cm) = 2.84 F cm^{-3} based on the volume of the device. By using Supplementary Equation 7, we obtain the areal energy density of the device where E_a = 1.60 F cm^{-2} × $(1\ V)^2$ / (2 × 3.6) = 222 μWh cm^{-2} while Supplementary Equation 8 enables calculation of a device's areal power density (P_a = 3.6 × 222 μWh cm^{-2} / 3194.1 s = 0.25 mW cm^{-2}). Metrics at higher scan rates and current densities (Supplementary Fig. 7g-i) as well as volumetric parameters are calculated with the same methods shown above.

Quasi-solid-state supercapacitor calculations: device structure is shown in Fig. 5a lower row and Supplementary Figure 9a. This device is a scaled down model for research purposes thus when calculating metrics of performance, we only consider basic components for the supercapacitor i.e., the electrodes and gel electrolyte. Other parts of the device such as Pt current lead, polyimide tape, epoxy sealing, and the reservoir for loading epoxy are not included. The thickness of the gel electrolyte layer is 0.07 cm, leading to a device thickness of 0.28 cm + 0.28 cm + 0.07 cm = 0.63 cm. The length and width of the device are the same as a single electrode i.e., 1 cm × 0.5 cm. The

weight of the device (two bricks + gel electrolyte) is 518 mg, leading to a PEDOT mass loading of 249 mg × 2.8% × 2 / 518 mg × 100% = 2.7%. Galvanostatic charge-discharge measurement at a current density of 0.5 mA cm^{-2} (Fig. 5c) is used for calculating capacitance, energy density and power density of a device. From the profile, the discharging time is 1736.2 s, leading to a q of 434.1 mC and obtained using Supplementary Equation 3 (0.5 mA cm^{-2} × 0.5 cm^{2} × 1736.2 s = 434.1 mC). Applying q to Supplementary Equation 1: C = 434.1 mC / 1 V = 434.1 mF. Using Supplementary Equations 4-6 and data from the third row of Supplementary Table 1 results in a C_a = 434.1 mF / 0.5 cm^{2} = 0.868 F cm^{-2}; $C_{g(brick)}$ = 434.1 / 499 mg = 0.87 F g^{-1} based on the mass of the device; $C_{g(PEDOT)}$ = 434.1 mF / (518 mg × 2.7%) = 31.0 F g^{-1} based on PEDOT mass; C_v = 434.1 mF / (1 cm × 0.5 cm × 0.63 cm) = 1.38 F cm^{-3} based on the volume of the device. By using Supplementary Equation 7, we obtain areal energy density of the device E_a = 0.868 F cm^{-2} × (1 V)^2 / (2 × 3.6) = 121 µWh cm^{-2}; using Supplementary Equation 8, we obtain areal power density of the device P_a = 3.6 × 121 µWh cm^{-2} / 1736.2 s = 0.25 mW cm^{-2}. Metrics at higher scan rates and current densities (Supplementary Fig. 9e-g) as well as volumetric parameters are calculated with the same methods shown above.

Supplementary Discussion

Estimation of capacitance and energy density delivered by a brick wall

Calculations are carried out assuming that a wall is constructed using standard bricks (20.32 cm × 10.16 cm × 5.72 cm) (Supplementary Fig. 12) stacked together and with a brick-brick separation gap of 1.43 cm (¼ of the brick's thickness); this gap is filled with a composition consisting of gel electrolyte and epoxy or cement. Our energy storing one-brick-thick hypothetical wall is constructed out of repeating units comprised of two stacked bricks that make up the anode and cathode. Each repeating unit has a length of 20.32 cm + 1.43 cm = 21.75 cm, thickness of 10.16 cm and height of 5.72 cm × 2 + 1.43 cm × 2 = 14.30 cm. In a repeating unit the area of PEDOT in contact with gel electrolyte is 20.32 cm × 10.16 cm × 4 = 825.80 cm^2. According to galvanostatic charge-discharge curves at 0.5 mA cm^{-2} (Fig. 5c), the areal capacitance and energy density of a nanofibrillar PEDOT-coated brick are 0.868 F cm^{-2} and 121 µWh cm^{-2}, respectively. Utilizing these numbers and applying them to our hypothetical wall, leads to a capacitance of 0.868 F cm^{-2} × 825.80 cm^2 / 2 = 358.40 F and energy of 121 µWh cm^{-2} × 825.80 cm^2 / 2 = 49.96 mWh for each repeating unit of the wall.

In order to provide a simple model for understanding the energy storage properties of our hypothetical wall, we have opted for describing energy storage in terms of a wall's front side area rather than the electrode's surface area. In this case, the repeating unit for the front side area of the wall is 21.75 cm × 14.30 cm = 0.03110 m^2 and a wall delivers an areal capacitance of 358.40 F ÷ 0.03110 m^2 = 11.5 kF m^{-2} and an energy density of 49.96 mWh / 0.03110 m^2 = 1.61 Wh m^{-2}. Note that our calculation is dependent on a brick's dimension and capacitance and areal energy density will increase if the front side area of the wall includes more bricks or if bricks are thicker. Note that our calculation is dependent on a brick's dimension and while holding front side area of wall

constant, capacitance and areal energy density will increase if the wall includes more bricks or if the wall is made up of bricks with wider dimensions. We believe that our brick wall will be ideal for powering embedded microdevices.
