# Supplementary material for: Energy storing bricks for stationary PEDOT supercapacitors
Source: Nat Commun. 2020 Aug 11;11:3882. doi: 10.1038/s41467-020-17708-1 (PMC7419536; doi:10.1038/s41467-020-17708-1)
Supplement: Supplementary file 3 — Description of Additional Supplementary Files [file 41467_2020_17708_MOESM3_ESM.pdf]

Description of Additional Supplementary Files

File Name: Supplementary Movie 1
Description: A tandem device comprised of three PEDOT-coated brick supercapacitors and connected in series lights up a white light-emitting diode for 10 min.
